# Supplementary material for: The missing link between genetic association and regulatory function
Source: eLife. 2022 Dec 14;11:e74970. doi: 10.7554/eLife.74970 (PMC9842386; doi:10.7554/eLife.74970)
Supplement: Transparent reporting form [file elife-74970-transrepform1.docx]

***eLife’s* transparent reporting form**

We encourage authors to provide detailed information *within their submission* to facilitate the interpretation and replication of experiments. Authors can upload supporting documentation to indicate the use of appropriate reporting guidelines for health-related research (see [EQUATOR Network](http://www.equator-network.org/  \t _blank)), life science research (see the [BioSharing Information Resource](https://biosharing.org/)), or the [ARRIVE guidelines](http://www.plosbiology.org/article/info:doi/10.1371/journal.pbio.1000412) for reporting work involving animal research. Where applicable, authors should refer to any relevant reporting standards documents in this form.

If you have any questions, please consult our Journal Policies and/or contact us: [editorial@elifesciences.org](mailto:editorial@elifesciences.org).

**Sample-size estimation**

- You should state whether an appropriate sample size was computed when the study was being designed
- You should state the statistical method of sample size computation and any required assumptions
- If no explicit power analysis was used, you should describe how you decided what sample (replicate) size (number) to use

Please outline where this information can be found within the submission (e.g., sections or figure legends), or explain why this information doesn’t apply to your submission:

**Replicates**

We use three types of data:

1. GWAS summary statistics.

2. Our calculated GWAS associations, using UKBB data, which have been used for many previous studies.

3. GTEx eQTL association data.

Each of these datasets has been previously used for the same purposes (GWAS, colocalization, and TWAS), so power has already been demonstrated by Chun et al. 2017, Giambartolomei et al. 2014, Hormozdiari et al. 2016, Gusev et al. 2016, GTEx Consortium et al. 2015, Mahajan et al. 2018, Liu et al. 2015, Goyette et al. 2015, and Zhang et al. 2020, all of which we cite.

Additionally, with each dataset, we used to maximum number of samples available while avoiding population stratification and relatedness.

- You should report how often each experiment was performed
- You should include a definition of biological versus technical replication
- The data obtained should be provided and sufficient information should be provided to indicate the number of independent biological and/or technical replicates
- If you encountered any outliers, you should describe how these were handled
- Criteria for exclusion/inclusion of data should be clearly stated
- High-throughput sequence data should be uploaded before submission, with a private link for reviewers provided (these are available from both GEO and ArrayExpress)

Please outline where this information can be found within the submission (e.g., sections or figure legends), or explain why this information doesn’t apply to your submission:

Our study does not run experiments, but instead is a new analysis of existing data, so the concept of experimental replication does not apply.

**Statistical reporting**

- Statistical analysis methods should be described and justified
- Raw data should be presented in figures whenever informative to do so (typically when N per group is less than 10)
- For each experiment, you should identify the statistical tests used, exact values of N, definitions of center, methods of multiple test correction, and dispersion and precision measures (e.g., mean, median, SD, SEM, confidence intervals; and, for the major substantive results, a measure of effect size (e.g., Pearson's r, Cohen's d)
- Report exact p-values wherever possible alongside the summary statistics and 95% confidence intervals. These should be reported for all key questions and not only when the p-value is less than 0.05.

Please outline where this information can be found within the submission (e.g., sections or figure legends), or explain why this information doesn’t apply to your submission:

(For large datasets, or papers with a very large number of statistical tests, you may upload a single table file with tests, Ns, etc., with reference to sections in the manuscript.)

The calculation for P-values displayed in supp. fig. 1 are explained in the “Enrichment analysis” section of the methods. Other reported P-values and posterior probabilities are obtained from the published methods used, which can be found in the cited papers Chun et al. 2017, Giambartolomei et al. 2014, Hormozdiari et al. 2016, Gusev et al. 2016, and Wang et al. 2020.

**Group allocation**

- Indicate how samples were allocated into experimental groups (in the case of clinical studies, please specify allocation to treatment method); if randomization was used, please also state if restricted randomization was applied
- Indicate if masking was used during group allocation, data collection and/or data analysis

Please outline where this information can be found within the submission (e.g., sections or figure legends), or explain why this information doesn’t apply to your submission:

**Additional data files (“source data”)**

Because we used phenotypes already recorded along with genomic information, we did not group or define subjects.

- We encourage you to upload relevant additional data files, such as numerical data that are represented as a graph in a figure, or as a summary table
- Where provided, these should be in the most useful format, and they can be uploaded as “Source data” files linked to a main figure or table
- Include model definition files including the full list of parameters used
- Include code used for data analysis (e.g., R, MatLab)
- Avoid stating that data files are “available upon request”

Please indicate the figures or tables for which source data files have been provided:

A table (data_for_fig3.tsv) has been provided listing the genes that were identified as hits with each method, as displayed in Fig. 3.
